# P53 protein and the diseases in central nervous system

**DOI:** 10.3389/fgene.2022.1051395

**Published:** 2023-01-09

**Authors:** Li Lei, Qixiong Lu, Guifang Ma, Tao Li, Jiahong Deng, Weijia Li

**Affiliations:** ^1^ The Affiliated Hospital of Kunming University of Science and Technology, The Department of Neurosurgery, The First People's Hospital of Yunnan Province, Kunming, Yunnan, China; ^2^ Department of Ear, Nose and Throat (ENT) and Head and Neck (HN) Surgery, The First People’s Hospital of Yunnan Province, The Affiliated Hospital of Kunming University of Science and Technology, Kunming, China

**Keywords:** P53, cerebral stroke, neurodegenerative diseases, gliomas, apoptosis, ferroptosis, neuroinflammation

## Abstract

P53 protein is the product of *P53* gene, which is a well acknowledged tumor suppressor gene. The function of P53 and the relevant mechanisms of anti-neoplasm have raised the interest of researchers since many years ago. It is demonstrated that P53 is a basic cell cycle regulator and a strong inhibitor for versatile cancers in humans. However, most research focuses on other organs and systems instead of the central nervous system (CNS). In fact, in recent years, more and more studies have been suggesting that P53 plays a significant role in multiple CNS tumors and other diseases and disorders such as cerebral stroke and neurodegenerative diseases. In this work, we mainly reviewed the P53’s relationship with CNS tumors, cerebral stroke and neurodegenerative diseases, together with the relevant mechanisms, aiming to summarize the research achievements and providing new insight to the future study on diseases in CNS.

## 1 Introduction

The *P53* gene, with the full name of *Tp53* gene, is a well acknowledged tumor suppressor gene and thus thoroughly and repeatedly studied in numerous cancer types all over the human body and even in other diverse mammals. Its protein product, with the molecular weight of 53 kDa, therefore got its name. The expression and structure of P53 is relatively constant and conservative in mammals and other organisms. For the past decades, researchers have proven that it is an important cell cycle checker and a cornerstone to impact almost all cancers in humans. This study reviewed the current knowledge we have acquired from P53 and its relationship with CNS diseases including gliomas, cerebral stroke and neurodegenerative diseases. In addition, the relevant mechanisms involving the regulation of apoptosis, ferroptosis and inflammation were reviewed and discussed.

Glioblastoma is the most common primary malignant and aggressive brain tumors in humans. The treatment for it, unfortunately, no progress can be seen till recent years. Whether it is surgical resection or combined with radiotherapy and chemotherapy, there has been no significant improvement in the prognosis of high grade or recurrent gliomas. It is well known that P53 can regulate apoptosis, thus theoretically it is able to inhibit the proliferation of gliomas (Hernández Borrero and El-Deiry, 2021). Though less reported, we believe that P53 may have the potential to play a role in the treatment of gliomas.

Cerebral stroke is clinically divided into two types: ischemic stroke and hemorrhagic stroke. The former has the highest incidence, and is due to the brain blood insufficiency or cut-off, resulting in brain tissue damage and massive neuronal deaths. Although hemorrhagic stroke has lower incidence compared to the ischemic one, it is with higher mortality and severer clinical outcome (Zhang et al., 2022). In stroke, reducing the death of neurons is a treatment plan that can improve the prognosis of patients (Zhang et al., 2022). By regulating the expression of P53 to increase the survival of neuronal cells, it might be used as a molecular-targeted treatment after stroke.

Neurodegenerative diseases are a cluster of diseases involving CNS and can be characterized by sensorimotor function impairment, memory loss and dementia, among which Alzheimer’s disease (AD), Parkinson’s disease (PD), Huntington’s disease (HD) and amyotrophic lateral sclerosis (ALS) are most common in clinical practice. The shared pathological changes in such diseases are atrophy of cerebral lobes, loss of neurons and synapses and accumulation of abnormal proteins in neurons or brain parenchyma such as amyloid beta (Aβ), tau protein and Lewy bodies. Neurodegenerative diseases are now considered to be incurable and the mechanisms behind are still unknown. However, *via* countless research, it is revealed that apoptosis, ferroptosis and neuroinflammation may play a fundamental role in them.

## 2 P53 structure

### 2.1 Wild-type P53 structure

The P53 protein structure includes five main parts: the reverse activation domain, the proline-rich domain, the DNA binding domain, the tetramer domain and the regulatory domain (Harris, 1996a).

The protein P53 has been proven to have transcription activation function that may be located at the amino terminal residues 1–42 (Lin et al., 1994). The reverse activation domain may be related to the tumor inhibitory function of P53, because the mutated P53 protein of many tumor patients have lost both transcription activation activity and tumor suppressor activity (Raycroft et al., 1990). This domain also can mediate the interaction between P53 and some basic transcription factors, like TATA-binding protein (TBP) (Liu et al., 1993).

The proline-rich domain has proved to have little to do with transcriptional activation, but the absence of this domain has weakened the ability of P53 to inhibit the growth of tumor cells *in vitro* (Walker and Levine, 1996). P53 proline-rich domain plays a key role in inhibiting signal transmission downstream of P53 protein and can be associated with signaling pathways transduction. Research shows that the domain participates in growth suppression cell signal transmission (Walker and Levine, 1996).

**FIGURE 1:**
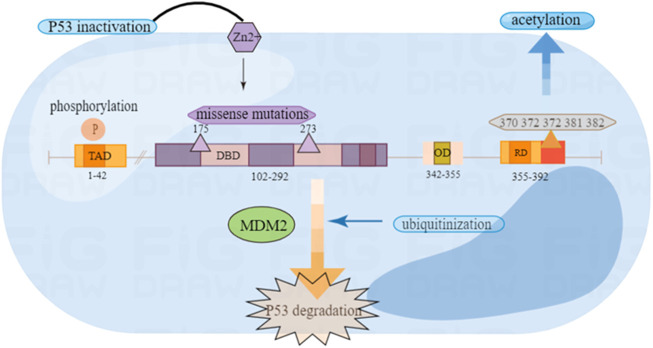
Wild type P53 structure and common mutation sites.

The P53 protein is a tetrameric transcription factor. Oligomerization appears to be essential for the tumor suppressing activity of P53 because oligomerization-deficient P53 mutants cannot suppress the growth of carcinoma cell lines (Jeffrey et al., 1995; Kato et al., 2003). The DNA binding domain (DBD) is key point of P53 for functioning. Experiments show *in vitro* that it is composed of the basis of two decamers RRRCWWGYYY (n) RRRCWWGYYY (R = purine, C = cytosine, W = adenine or thymine, G = guanine, Y = pyrimidine, and n means 0–13 bases) (Jordan et al., 2008; Hernández Borrero and El-Deiry, 2021). Through this domain, P53 acts as a transcription factor in a sequence-specific way by identifying the P53 reaction element (el-Deiry et al., 1992). Another area is the regulatory domain (RD), which can combine with PUMA and MDM2 to produce corresponding changes (Figure 1).

### 2.2 Common structure mutation of P53

In P53 mutants, missense mutations are one of the most common mutation types, which are divided into DNA contact mutants and structural mutants. The first type of mutant, such as the P53-R273H mutant, can be replaced with residues in direct contact with DNA (Hainaut and Pfeifer, 2016). This kind of mutant may not shake the structure of P53. However, it may destruct the heat-stability. The mutant is characterized by the core unfolding area compared to the wild type. Another type of mutation is amino acid substitution that can destroy the core folding area, such as the P53-R175H mutation, which will lead to changes in the structure of P53. In humans, the replacement of codons 175 and 273 is one of the most common mutations (Bykov et al., 2018). In previous experiments, it has suggested that the mutations of R175H and R273H subverted the ability to bind zinc ions, and the function of P53 can be restored after treatment with zinc ion metallochaperones (Garufi et al., 2013). The mutated P53 will combine with wild P53 to make it inactive and no longer have tumor inhibitory function (Hernández Borrero and El-Deiry, 2021) (Figure 1).

## 3 Regulation of P53

As is known, the common post-translation modifications (PTM) include: phosphorylation, acetylation, ubiquitinization, methylation, demethylation and glycosylation (Hernández Borrero and El-Deiry, 2021). These modifications play an important role in stabilizing and activiting P53 (Brooks and Gu, 2003).

### 3.1 Phosphorylation

Phospholation is a modification of the P53 protein after translation, which can be phosphorylated on multiple amino acids in the N-terminal transcriptional activation area of the protein (Nakamizo et al., 2008). Post-translational modification of P53 through phosphorylation is considered to be an important mechanism to regulate the stability and function of P53. Moreover, the phosphorylation of P53 plays an important role in regulating activities such as DNA binding (Ashcroft et al., 1999). Most importantly, the phosphorylation of P53 plays an important role in regulating its activity. For example, it can actively regulate the transcriptional activity of many transcription factors, including c-jun, CREB and NF-IL6/LAP, and regulate the stability of other proteins such as c-jun and NF-κB. Several serine residues of P53 protein in amino and carboxyl end regions are phosphorylated by some cytokinases (Unger et al., 1999). Phosphorylation and acetylation of P53 can interrupt negative regulatory factors (Chao et al., 2006).

The experimental results show that the simulated structural phosphorylation P53 constructor (Ad-P53-18D20D) at Thr18 and Ser20 can induce G1 stagnation of normal cell strains after exogenous non-phosphorylation P53 (Ad-P53) administration, indicating that the phosphorylation of P53 at Thr18 and Ser20 is enough to induce P53-mediated glioma cell apoptosis (Nakamizo et al., 2008). Experiments have also shown that Ser18 and 23 phosphorylation is required for P53-dependent apoptosis and tumor suppression in mice (Chao et al., 2006). Therefore, the regulation of phosphorylation after P53 translation may offer a potential treatment strategy in the treatment of tumors.

### 3.2 Ubiquitinization

Ubiquitin is a polypeptide composed of 72 amino acids. It can modify the translated proteins, and the most common function of ubiquitin modification is to target the degradation of substrate protein through the proteasome. The covalent connection process of ubiquitin is called ubiquitinization (Pickart, 2001). MDM2 is a ubiquitin ligase and exhibits the negative regulating effect on P53 (Watson and Irwin, 2006). Both MDM2 and MDMX can negatively regulate the transcription activity and stability of P53. MDM2 is the target of P53 transcription. As a ubiquitin ligase, P53 is ubiquitinized by MDMX, which is a kind of PTM that damages the function of P53 (Joerger and Fersht, 2016; Kwon et al., 2017). Therefore, blocking the interaction between P53 and MDM2 can stabilize it, leading to the cessation of the cell cycle and preventing the further development of the tumor. It also provides strategies for molecular treatment of tumors (Vassilev et al., 2004). MDM2 is functioning in three pathways, as the negative regulator of P53. The first is to combine P53 with its activation area to inhibit its transcription ability (Linke et al., 2008). The second is to participate in the nuclear output of P53. Third, it promotes the degradation of P53 as a ubiquitin ligase (Michael and Oren, 2003). Some scholars used small-molecule inhibitor to suppress the MDM2-P53 complex and proved that in mouse, P53 could be activated and tumor growth be suppressed (Vassilev et al., 2004; Zhao et al., 2015). Experiments show that MDM2 antagonists promote cell growth inhibition and apoptosis, which brings potential value to the treatment of tumors (Tortora et al., 2000).

### 3.3 Acetylation

Acetylation can modify the lysine (K) residue at C-terminal of P53, which is an important modification site after the transcription of P53. The C-terminal regulation domain of six lysine residues of P53 (K370 K372 K373 K381 K382 K386) can be targeted by MDM2. Acetylated modification can cause the transcription activity of P53 to be activated and increase its stability. CBP/p300 (p300 is the auxiliary activator required for P53-dependent Waf1/Cip1 transcription activation) is a transcriptional co-activator protein, which can interact with P53 to regulate the early cell cycle and trigger apoptosis of genetically intoxicated cells. So as to prevent the further development of the tumor (Lee et al., 1998; Grossman et al., 2003). After DNA damage, P53 can also be protected from degradation through the acetylation targeting on carboxyl terminal. This acetylation is conducive to cell survival because it promotes the expression of cell cycle stagnation target genes controlled by P53, such as the cell cyclin-dependent kinase inhibitor 1A (CDKN1A, also known as P21) (Knights et al., 2006; Kruse and Gu, 2008). Moreover, there are K120 and K164 in the binding domain of P53 DNA, which are the most common mutation regions of P53 in solid malignant tumors. For example, in glioblastoma, K164 has a mutation, indicating that acetylation of P53 plays a significant role in tumor inhibition (Zhang et al., 2015; He et al., 2017).

## 4 Regulation target of P53

### 4.1 PUMA and P53

P53 upregulated modulator of apoptosis (PUMA) was identified as a transcription target for P53 (Nakano and Vousden, 2001). It is highly conservative between humans and mice. The genetic structure of PUMA in mice and humans is also similar (Han et al., 2001). This protein belongs to the BH3-Only subgroup of Bcl-2 protein family. The BH3 domain of PUMA is necessary for its interaction with Bcl2-like proteins (Naik et al., 2007). The BH3 domain of PUMA forms a bikinetic alpha-helical structure, which binds directly to the anti-apoptotic Bcl2 family (Day et al., 2008). The C-terminal part of PUMA contains a hydrophobic domain to guide its mitochondrial location. BH3 domain and mitochondrial localization are essential for PUMA’s ability to induce apoptosis or inhibit cell survival (Yu and Zhang, 2008). PUMA is usually expressed at a low level, but once stressed, its expression will be immediately induced (Yu et al., 2001). Bioinformatic analysis revealed the gene promoters, exons and introns of PUMA. In the transcription factors of PUMA, P53 plays a significant role, and its function is explored most thoroughtly for now.

PUMA is a member of the Bcl-2 family that only relies on BH3 (BH3). It is an important mediator of P53-dependent and independent apoptosis. It transmits death signals to mitochondria, where it indirectly acts on Bcl2 family members by removing the inhibition exerted by anti-apoptosis members. Bax and/or Bak. It directly binds and antagonizes all known members of the Bcl2 family who are anti-apoptotic, thus inducing mitochondrial dysfunction and caspase activation. Therefore, PUMA can be activated to inhibit tumor growth by restoring the apoptosis of cancer cells (Yu and Zhang, 2008). In this process, the promoter of PUMA binds to P53 to promote the modification of core histones. For example, as is mentioned above about the post-translational acetylation, P53 acetylates the core histones, leading to the opening and transcriptional activation of the chromatin structure. After P53 activates PUMA, it initiates cell apoptosis, thus preventing tumor cells from growing (Kim et al., 2019). To sum up, after DNA damage occurs, nuclear P53 will immediately induce PUMA production, thus protmoting apoptosis.

Zhang et al. reported in their study that the role of miR-221/222 in the regulation of apoptosis was confirmed. MiR-221/222 gene knockout (KO) can cause mitochondrial membrane potential changes and caspase-mediated apoptosis. In addition, they also proved that the apoptotic protein PUMA is negatively regulated by miR-221/222 (Zhang et al., 2010). In glioblastoma, experiments show that miR-221/222 regulates the mitochondrial pathway by directly targeting PUMA to induce cell survival (Zhang et al., 2010).

### 4.2 P21 and P53

Similar to PUMA, CDKN1A (P21) is also one of the downstream factors regulated by P53. It is one of the important target genes for P53 to induce cell cycle stagnation. *Cdkn1a* gene encodes P21W AF1 protein. AF1 protein is a cyclin-dependent kinase inhibitor that can directly interact and inhibit the cyclin-dependent kinase (CDK) complex, causing cell cycle stagnation (el-Deiry et al., 1993; Harper et al., 1993). Through the action of CDK, P21 inhibits the phosphorylation of Rb, and then Rb binds to E2F to prevent the transcription required for the progression of the cell cycle. P21 can interact with multiple CDK complexes expressed in the cell cycle, resulting in the cell cycle stagnation in different phases. Specifically, the interaction between P21 and Cyclin E/CDK2 and Cyclin D/CDK4 promotes the binding of Rb to E2F, leading to the cease in G1 phase (Nakanishi et al., 1999; Stewart et al., 1999). On the other hand, the binding of P21 to Cyclin B/CDK1 leads to G2/M cell cycle stagnation (Dash and El-Deiry, 2005). Therefore, as a downstream factor of P53, P21 can also participate in the regulation of the cell cycle.

## 5 P53 and gliomas

Tumors arising from glial cells (gliomas) make up the most common group of primary brain tumors (Friedmann-Morvinski et al., 2012). Despite the use of multiple therapies in combination regarding surgery, radiotherapy and/or chemotherapy, the survival of patients with high grade or recurrent gliomas remains poor; the median survival of patients with glioblastoma is less than a year (Phillips et al., 2006; Ma et al., 2021). In the latest WHO glioma classification, adult-type diffuse gliomas are divided into the following categories: 1) Astrocytoma, IDH-mutant; 2) Oligodendroglioma, IDH-mutant, and 1p/19q-codeleted; 3) Glioblastoma, IDH-wild type (Louis et al., 2021). To improve therapeutic approaches for patients with gliomas and to understand glioma biology better, current research has focused on molecular and genetic alterations associated with the development and progression of gliomas (Ma et al., 2021).

The *P53* gene, which resides on chromosome 17p13.1 and encodes the P53 protein, is the most frequent target for mutations in human cancers, with more than half of all tumors exhibiting a mutation at this locus (Vogelstein et al., 2000; Freed-Pastor and Prives, 2012). Loss of *P53* transcriptional activity, mutations in *P53* gene or inhibition on P53 signaling, are major contributing factors to malignant transformation (Jain and Barton, 2018). P53 participates in many cellular functions including cell cycle control, DNA repair, cell differentiation, genomic plasticity, and programmed cell death (Hollstein et al., 1991; Harris, 1996b). The prime function of wild type P53 is the ability to promote the stagnation of the cell cycle and apoptosis (Kastenhuber and Lowe, 2017). Therefore, the activation of P53 can prevent and eliminate DNA-damaged cells to prevent the accumulation of oncogene mutations to ward off cancer (Livingstone et al., 1992). P53 also functions to modulate the downstream signaling pathway or modify the translated protein to regulate the apoptosis of tumor cells and cell cycle (Brady et al., 2011).

The glioma is the most common primary brain tumors in neurosurgery, especially glioblastoma (GBM) which is with the highest grade. It usually has a fully structured *P53* gene. Therefore, the proliferation of GBM and resistance to treatment may be related to the loss of P53 function (Lou et al., 2020). In the experiment, cyclic RNA CDR1as is widely expressed in the brain of mammals, which can decrease with the increase of glioma grade and can predict the total survival period of patients with glioma. Moreover, CDR1as can bind to and stabilize P53 by preventing ubiquitination. CDR1as interacts directly with the P53 DBD domain necessary for MDM2 binding, thus undermining the formation of the P53/MDM2 complex (Lou et al., 2020), preventing MDM2 from negatively regulating P53. The study also proved that the enhanced expression of CDR1as significantly inhibits cell proliferation, while the down-regulation of CDR1as promotes cloning (Lou et al., 2020).

Not only cyclic RNA CDR1as can inhibit cell proliferation by changing the relationship between MDM2 and P53. In one experiment, miRNA-129 inhibits glioma cell growth by targeting CDK4, CDK6, and MDM2. The experimental results show that over-expression of miR-129 can reduce the expression of CDK4 genes in HEK293 cells by 58.9% and CDK6 by 35.7%. The expression of MDM2 has also been reduced by 49%. Therefore, we can say that miR-129 significantly targets and inhibits the expression of CDK4, CDK6 and MDM2. Additionally, miR-129 also inhibits cell proliferation by affecting MDM2 (Moradimotlagh et al., 2020).

**FIGURE 2:**
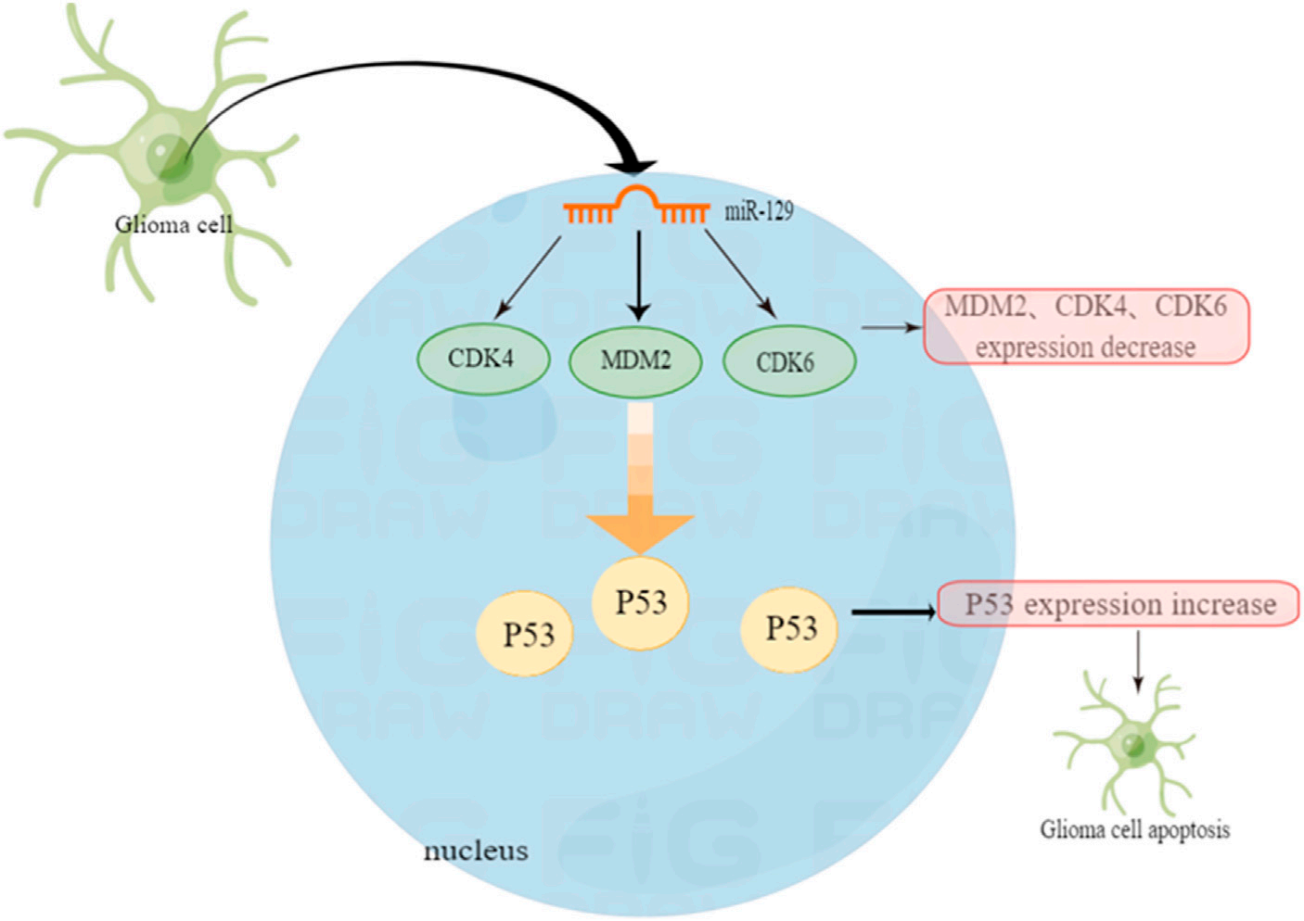
P53 function in glioblastoma and the regulation mechanism of non-coding micro RNA.

In another experiment, it was also confirmed that miR-29a can raise the level of P53 and induce apoptosis dependent on P53 function (Park et al., 2009). Chen et al. reported that miR-29a negatively regulates the expression of MDM2 by directly targeting MDM2 in glioma cells (Chen et al., 2021). As a negative regulator of the *P53* gene, over-expression of miR-29 can precisely inhibit MDM2. That is to say, it can enhance the stable expression of P53, thus facilitating apoptosis and cell cycle stagnation in tumor cells (Moradimotlagh et al., 2020). The various MDM2 inhibitors mentioned above have shown inhibitory effects on MDM2 and P53, laying the foundation for future treatment of glioma and improvement of its prognosis. For example, the MDM2 inhibitor RG7112, the first one to be admitted to clinical trials, although not yet extensively carried out, it provides a certain basis for molecular targeted treatment of gliomas (Figure 2).

## 6 P53 and cerebral stroke

### 6.1 P53 and ischemic stroke

Ischemic stroke is the main cause of morbidity and death, and the result depends on the extent on the number of neuronal deaths. Cerebral ischemia and hypoxia activate P53 to provide targets for the treatment of stroke (Arumugam et al., 2018). Many studies have shown that P53 is an apoptosis-promoting factor, which expresses an increase in cell apoptotic injury after cerebral ischemia. In addition, in different stroke models, the loss of P53 or the application of P53 inhibitors may potentially reduce the volume of cerebral infarction (Hong et al., 2010). Not only that, more and more experiments have shown that P53 is related to the death of neurons in animal experimental models of cerebral ischemia or hypoxia (Banasiak and Haddad, 1998; Leker et al., 2004; Endo et al., 2006; Yonekura et al., 2006; Damisah et al., 2020). P53-mediated apoptosis is a common cell death mechanism that can be triggered by oxidative stress or DNA damage (Amundson et al., 1998). It is activated in the cerebral ischemia area and promotes the apoptosis of neurons. The lack of P53 or the application of its inhibitors can significantly reduce brain damage. P53-mediated apoptosis of nerve cells occurs through a variety of molecular mechanisms, such as Notch signaling pathway (Hong et al., 2010; Arumugam et al., 2018). Notch1 is a membrane receptor that regulates the proliferation, differentiation and transition of cells in a series of tissues (Lathia et al., 2008). In the developing brain, Notch signals participate in the preservation of nerve precursor cells in an unduplicated state, partly by inhibiting neurogenesis. Notch signals also affect synaptic plasticity and learning memory in the adult brain (Alberi et al., 2013; Sargin et al., 2013). As shown in a study, active Notch inhibits the growth of B-cell, interrupts the cell cycle, and induces apoptosis (Morimura et al., 2000). An active form of Notch1 can raise the level of nuclear P53 to promote the transcription of apoptosis genes (Yang et al., 2004). Notch1 signaling pathways and four important interaction pathways (NF-κB, P53, HIF-1α and PIN1) are aggregated on a conservative DNA-related nucleopolyprotein complex that control the expression of genes that determine the fate of neurons. In mice experiments, mice with Notch inhibitors showed reduced damage to brain cells and improved functional results. Therefore, inhibiting Notch may prevent P53-mediated apoptosis and improve the activity of neurons (Arumugam et al., 2018).

Similarly, in the model of simulating cerebral ischemia, mouse focal cerebral ischemia, global cerebral ischemia (GCI) and transient frontal-temporal ischemia, it is found that the mRNA and protein of P53 in the ischemic area are elevated (van Lookeren Campagne and Gill, 1998; Watanabe et al., 1999; Hong et al., 2010). Research also shows that not only does the expression of P53 increase in ischemic areas, but also in astrocytes and neuron cells (Banasiak and Haddad, 1998). In ischemic stroke, the damage of cerebral endothelial cells is caused by ischemia. In order to prove the role of activated protein C (APC) in stroke, a hypoxic cerebral endothelial cell (CEC) injury model is constructed (Cheng et al., 2003). APC is a systemic anticoagulant and anti-inflammatory factor which can reduce organ damage. Experiments show that APC directly prevents the apoptosis of hypoxic human endothelial cells by suppressing P53 at transcriptional level, normalizing the Bax/Bcl2 ratio that promotes apoptosis and reducing the Caspase-3 signal transduction (Cheng et al., 2003).

**FIGURE 3:**
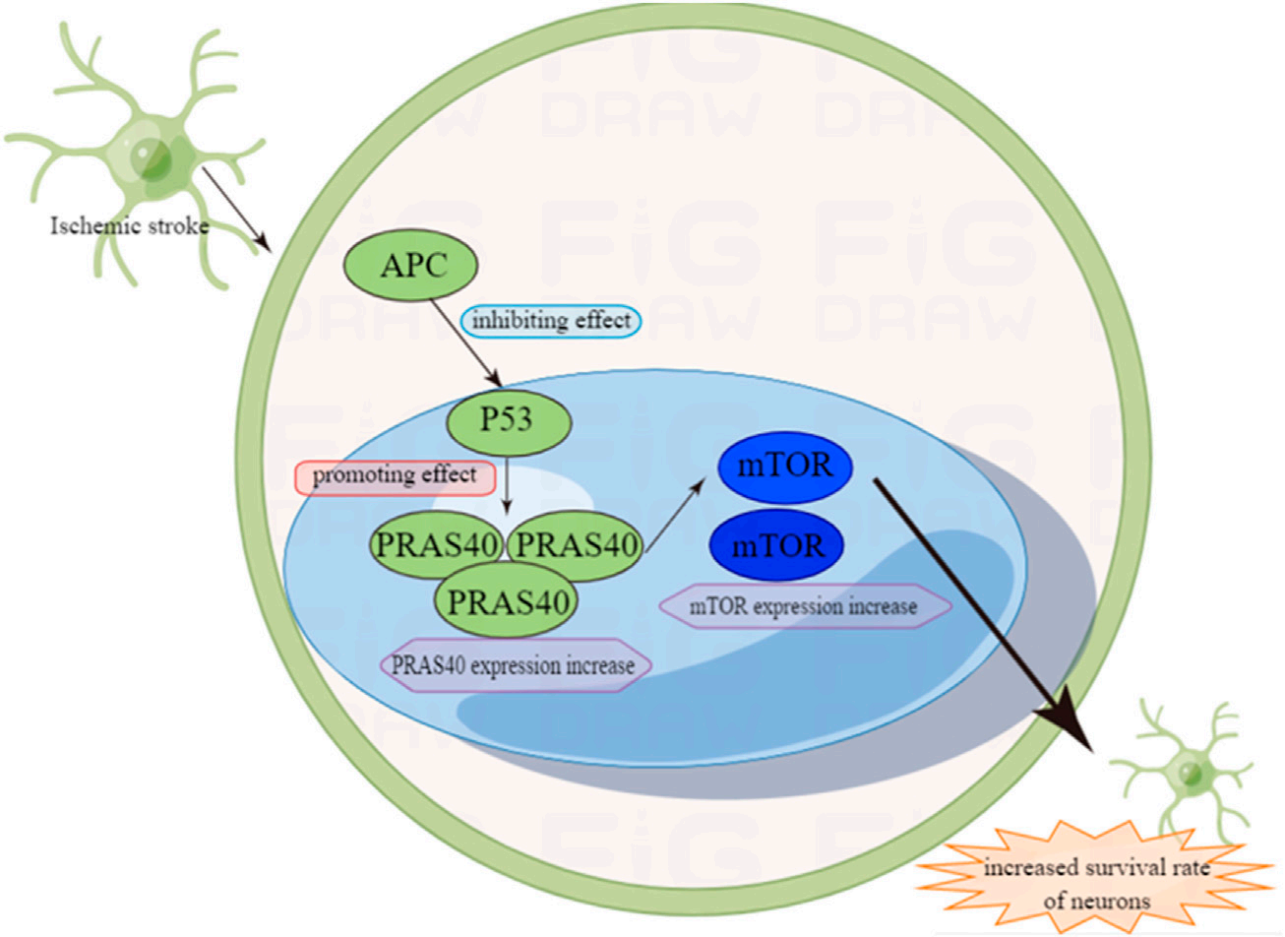
The role of P53 in ischemic stroke.

P53 can also regulate ischemic stroke through the P53/PRAS40/mTOR pathway. The mTOR pathway is involved in a variety of physiological processes, including cell metabolism, growth, differentiation, development and cell survival (Liu and Sabatini, 2020). In addition, it is also involved in the protection of cerebral ischemia (Xie et al., 2018). In previous studies, it has been proven that the activation of mTOR can reduce stroke-related neurons (Xie et al., 2014). In the P53/PRAS40/mTOR pathway, one of the important components of the mTOR complex is the proline-rich Akt and substrate PRAS40, which is at downstream of Akt, and the phosphorylated PRAS40 (pPRAS40) can activate the mTOR pathway (Kovacina et al., 2003; Wiza et al., 2014). Previous studies have reported the negative feedback relationship between P53 and PRAS40, indicating that P53 can be inhibited by its downstream factor PRAS40. Therefore, it can connect P53 with PRAS40 and mTOR and play an important role in ischemic stroke (Xiong et al., 2014). The brain damage of mice with gene *PRAS40* knockout is more serious than normal mice after ischemic stroke. The over-expression of pPRAS40 can reduce brain ischemia/reperfusion (I/R) damage and autophagy by activating mTOR. Not only that, studies have suggested that there is a negative feedback relationship between P53 and PRAS40, and P53 can be inhibited by its downstream factor PRAS40 (Havel et al., 2015; Zhao et al., 2021). In three groups of mice experiments: *P53* KO mice (*P53*−/−), heterozygous (*P53*+/−) mice, and WT mice (*P53*+/+), the results showed that both P53 KO and heterozygous groups had an improved neurological function and reduced area of cerebral infarction, and P53 KO group showed a better protective effect (Havel et al., 2015). This study indicates that I/R damage to neurons *in vivo* and *in vitro* can be reduced by inhibiting the P53/PRAS40/mTOR pathway (Figure 3).

### 6.2 P53 and hemorrhagic stroke

P53 can regulate hemorrhagic stroke by indirectly regulating ferroptosis. Ferroptosis is a non-apoptotic programed cell death which has been discovered in recent years (Zhang et al., 2021). Being different from apoptosis, ferroptosis is triggered by iron overload intracellularly, and characterized by specific pathological changes such as unique mitochondrial shrinking under transmission electronic microscope (TEM) (Alim et al., 2019). It has been noted that P53 has a strong relationship with ferroptosis especially in cerebral stroke (Jiang et al., 2015).

In ferroptosis, the three most important components are lipids, iron and ROS. Metabolic disorder of one of the three may lead to ferroptosis. P53 participates in the metabolic regulation of the three, therefore we have good rationale to infer that P53 can regulate ferroptosis (Liu and Gu, 2021).

Ferroptosis can be driven by fatal lipid peroxidation, which may be caused by cell metabolism and redox imbalances (Jiang et al., 2021). In 1950s, studies showed that cysteine deficiency may cause cell death, and endogenously synthesized cysteine may resist cell death (Coltorti et al., 1956; Eagle et al., 1961). Cysteine is a speed limiting factor in synthesis of glutathione (GSH), which can pass through neutral amino acid transporter or oxidized by cysteine/glutamate reverse transporter (a transmembrane protein complex containing SLC7A11 and SLC3A2 subunits, known as system xc−) (Bannai and Kitamura, 1980; Sato et al., 1999) to absorbed in environment or synthesized using the sulfurization pathway of methionine and glucose (Meister, 1995). GSH is a cofactor of many enzymes, including glutathione peroxidase (GPX4) and glutathione-S-transferase (Meister, 1995).

It has been reported that GSH and GPX4 can protect cells from various oxidative stress (Liu and Gu, 2021). GPX4 is a selenium protein that can reduce oxidized lipids. In the experiment from Conrad et al., the mouse model that knocked out the gene *Gpx4* showed that the deletion of the *Gpx4* gene would lead to the death of non-apoptotic cells realted to lipid peroxidation in mouse embryonic fibroblasts (Seiler et al., 2008). Therefore, inhibiting the lipid peroxidation of cells can reduce ferroptosis.

A study in 2015 demonstrated that P53 can promote ferroptosis by inhibiting the ability of cysteine to enter target cells (Jiang et al., 2015). In terms of mechanism, P53 can prevent cysteine from further generating GSH by inhibiting the transcription of subunit SLC7A11 of the cysteine/glutamate reverse transporter core. GSH is an antioxidant utilized by GPX4 to inhibit ferroptosis. Therefore, the inhibition of SLC7A11 by P53 can reduce intracellular GSH levels and promote cell ferroptosis (Yang et al., 2014).

It is well know that after intracerebral hemorrhage (ICH), one of the mechanisms causing brain cell death is *via* ferroptosis (Karuppagounder et al., 2016). Once ICH occurs, an increase in lipid peroxidation can be observed in neuronal cells (Karuppagounder et al., 2018). In mice experiments, the level of GPX4 in neurons is selectively manipulated to verify whether improving GPX4 has a protective effect on neurons after ICH. The results show that in the first 2 weeks of ICH, increased expression of GPX4 can reduce apoptosis, and have a protective effect on neurons (Alim et al., 2019).

**FIGURE 4:**
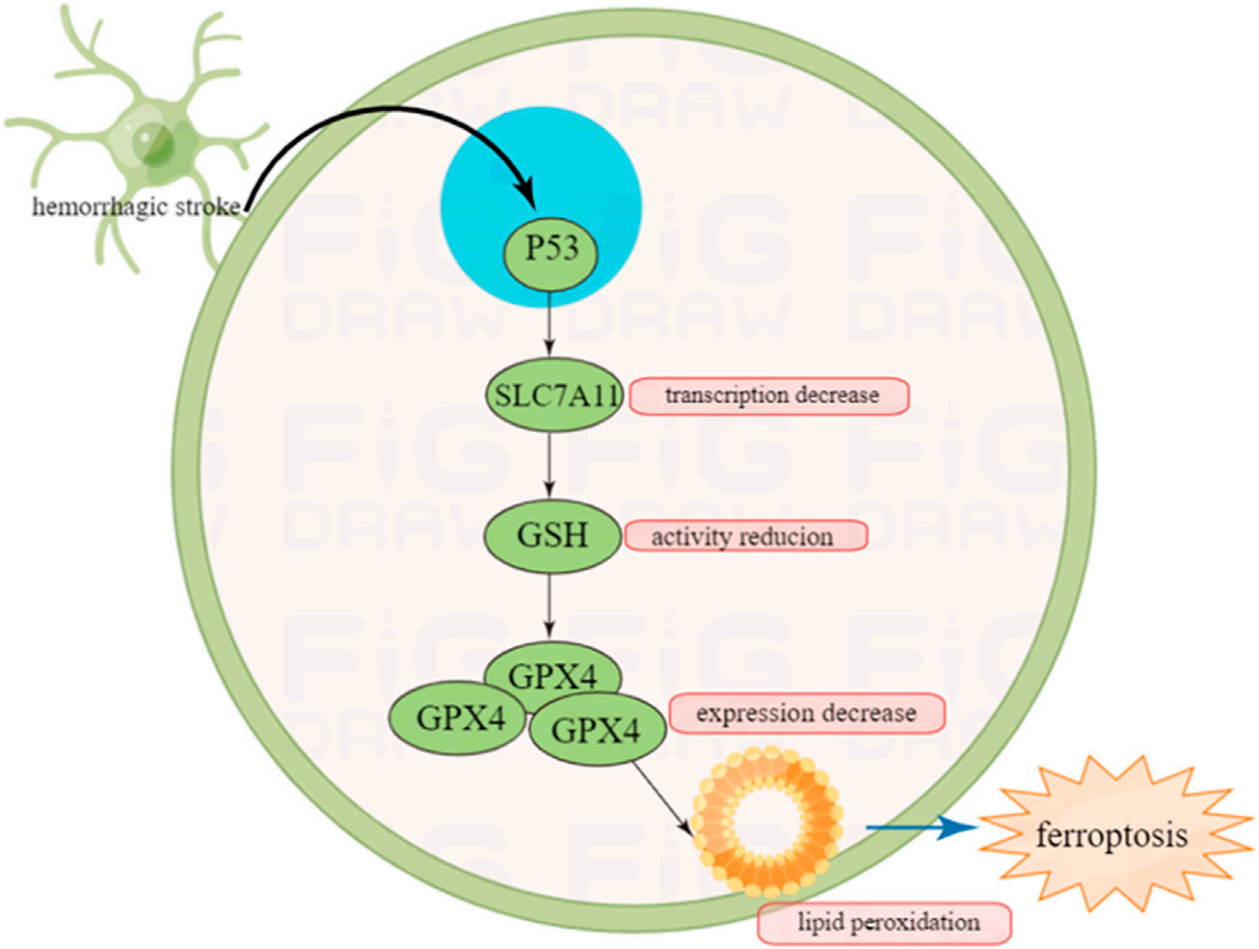
Inhibition of P53 on ferroptosis after hemorrhagic stroke and this may provide new insight in the treatment of cerebral stroke in the future.

To sum up, P53 may indirectly regulate the prognosis of intracranial hemorrhage by indirectly regulating ferroptosis, which also provides a certain basis for the treatment of hemorrhagic stroke (Figure 4).

## 7 P53 and neurodegenerative diseases

### 7.1 P53 and Alzheimer’s disease

Alzheimer’s disease (AD) is the most common age-related neurodegenerative disease (Olivier et al., 2010). Its clinical characteristics are progressive memory disorders, personality and behavioral changes (Qi et al., 2019). Its pathology is characterized by three main histological lesions, the senile plaques, the neurofibrillary tangles and atrophy of cerebral cortex. While senile plaques are extracellular deposits mainly due to the aggregation of a set of various hydrophobic peptides under the term of amyloid-β (Αβ) peptides (Checler and Alves da Costa, 2014). Αβ protein may cause neurotoxicity *via* inflammatory responses, especially small and soluble Aβ oligomers. The main pathological characteristics of AD are diffuse atrophy of the cerebral cortex and massive synaptic loss and excessive phosphorylated tau protein aggregates in cells (Qi et al., 2019), which is considered to be associated with many neurodegenerative diseases, and changes in tau protein are believed to be the result of downstream Αβ protein toxicity in the amylosis hypothesis (Dickson et al., 2013).

Due to the lack of timely access to anatomical tissue and various technical limitations, the death of nerve cells from apoptosis has always been controversial, but there is also some evidence that cell death in the brain of AD patients occurs to some extent through apoptosis (Kusiak et al., 1996). Some observations show that the decomposition and metabolism derived from β-Amyloid precursor protein (β-APP) can indeed trigger apoptosis in cell and animal models (Matsui et al., 2006). β-APP-derived fragments can also adjust P53. In order to identify P53 as a marker of AD, the experiment compared the impact of AD on the brain and the P53-like immune response in the control group. Through measurement, it was found that the expression of P53 was significantly enhanced, not only in glial cells, but also in many neuronal cells. This shows that P53 may lead to cell death in the AD brain (Ohyagi et al., 2005). Parallel increase in P53 and cell death can be observed in AD-affected brains to suggest that P53 plays a role in cell cycle stagnation, DNA repair and apoptosis (Bourdon et al., 2003; Aylon and Oren, 2011). Therefore, enhancing the expression of P53 may be the cause of an increase in cell death observed in the AD brain. P53 can also control the expression of proteins involved in AD pathology, indirectly affecting the course of the disease, rather than the classical target proteins.

P53 is also an important regulator for the expression of miRNAs. Some miRNAs, especially miRNAs from the miR-34 family, have been identified as direct transcription targets for P53. The expression of P53 horizontally induces the expression of miR-34 after transcription by binding to the DROSHA complex (Rokavec et al., 2014). In particular, miR-34a has the highest expression in the brain. As was reported, miR-34a could regulate the differentiation of neurons and the growth of axons. In the brain tissue of AD patients, it was found that its expression was up-regulated. John R. Dickson’s study found that changes in the expression of miR-34a can regulate the expression of tau in the pathogenesis of AD. The possible mechanism is that when the protein of tau aggregates, the up-regulation of miR-34a may be used as a compensation mechanism to reduce the expression of tua. This maight offer the opportunity to improve the prognosis of AD patients (Dickson et al., 2013). The miR-34a also regulates the expression of several synaptic proteins in cortical neurons (Jazvinšćak Jembrek et al., 2018). Oxidative stress is one of the conditions under which AD occurs. Under the condition of oxidative stress, P53 will react (Jazvinšćak Jembrek et al., 2018). Generally speaking, in mild oxidative damage, P53 can antioxidate to promote cell survival, while in severe oxidative stress, P53 can promote oxidative activity and lead to cell death (Jazvinšćak Jembrek et al., 2018). In human AD, the increase in the expression of P53 is directly proportional to the accumulation of Αβ protein in cells. Oxidative stress is closely related to mitochondrial dysfunction, and P53 can protect mitochondrial function by promoting the production of new mitochondria (Jazvinšćak Jembrek et al., 2018).

### 7.2 P53 and Parkinson’s disease

Parkinson’s disease (PD) is a motor-related disease characterized by the loss of dopamine energy neurons in the dense part of the grey matter (Pariyar et al., 2017). Oxidative stress and mitochondrial dysfunction are the main pathological motivations of PD. Slow motion, stiffness, tremor and abnormal posture are clinical manifestations (Talebi et al., 2021). The treatment of PD is drug therapy and surgical operation, mainly for symptoms. In autopsy studies, the expression of P53 and its target gene Bax in the tissues of PD patients increased (Qi et al., 2016). Recent literature shows that P53 is involved in the occurrence of familial PD through Parkin-mediated transcription regulation. Experiments show that the toxicity of α-synaptic nucleoprotein associated with P53 is shown in the PD model *in vitro* and *in vivo* (Duplan et al., 2016). P53 is also involved in the regulation of DJ-1, Park7’s mRNA and protein expression. P53 is a feasible missing link between hereditary and sporadic PD (Venderova and Park, 2012).

Parkin is a ubiquitin E3 ligase, a transcription inhibitor of P53. Exogenous *Parkin* gene deletion leads to increased *P53* gene expression (da Costa et al., 2009). Phorbol-12-myristate-13-acetate-induced protein 1 (PMAIP1/NOXA) balances the P53-dependent apoptosis associated with mitochondrial injures (Talebi et al., 2021). P53 can bind to Bcl2 to induce apoptosis by releasing Bcl2-associated protein X/Bcl2 homologous antagonist (Bax/Bak) from MPTP (Mattson and Magnus, 2006). The over-expression of P53 weakens the calcium ion transfer of mitochondria and provocates mitochondrial disturbance (Moon and Paek, 2015). Therefore, mitochondrial dysfunction caused by excessive expression of P53 is an important factor in PD progression. Moreover, the interaction of P53/Bclxl destroys the mitochondrial membrane potential and participates in the pathogenesis of PD.

## 8 Discussion and perspective

As a classic oncogene, P53 has guiding significance in tumor treatment and CNS diseases. In molecular biology, it provides a theoretical basis for treatment. For example, in glioblastoma, surgical operation plus radiotherapy and chemotherapy do not significantly improve the 5-year survival rate; the regulation of tumor cell death by P53 can be used as a potential strategy to improve the outcome of glioblastoma. Research on P53 has not been interrupted, and it has also been found that more and more molecules are regulated or controlled by P53, and *vice versa*. This has laid a solid foundation for the future development of drugs and molecular targeted treatment. However, there are relatively few studies of P53 in cerebral ischemic and hemorrhage stroke, particularly hemorrhagic stroke. After hemorrhagic stroke, how to reduce the apoptosis of neurons and glial cells through the regulation of P53 is an important subject in the future. Regulation of the expression of P53 to treat cerebral hemorrhagic stroke may become a potential hotspot.
